# Supplementary material for: Burst-tree decomposition of time series reveals the structure of temporal correlations
Source: Sci Rep. 2020 Jul 22;10:12202. doi: 10.1038/s41598-020-68157-1 (PMC7376115; doi:10.1038/s41598-020-68157-1)
Supplement: Supplementary file 1 — Supplementary information [file 41598_2020_68157_MOESM1_ESM.pdf]

Supplementary Information of “Burst-tree decomposition of time series reveals the structure of temporal correlations”

Hang-Hyun Jo*

Department of Physics, The Catholic University of Korea,

Bucheon 14662, Republic of Korea and

Asia Pacific Center for Theoretical Physics, Pohang 37673, Republic of Korea

Takayuki Hiraoka

Department of Computer Science, Aalto University, Espoo FI-00076, Finland and

Asia Pacific Center for Theoretical Physics, Pohang 37673, Republic of Korea

Mikko Kivelä

Department of Computer Science, Aalto University, Espoo FI-00076, Finland

* h2jo@catholic.ac.kr

I. CASES OF MERGING MORE THAN TWO BURSTS

In the main text, we have only considered a binary tree for the burst-tree decomposition method. However, in reality there can be "non-binary" cases where more than two bursts are to be merged at the same time. For example, three consecutive bursts separated by the interevent times (IETs) of the same length will be merged into one burst as soon as the time window Δt exceeds those IETs. Whenever we have such a situation, we record the number of bursts to be merged at the same time, denoted by $n_{\rm nb}$, and the value of IETs separating those bursts, denoted by $\tau_{\rm nb}$. From the event sequence of the editor 1, we obtain the frequency of $n_{\rm nb}$ for all cases, as shown in Fig. S1(a). Then for each $n_{\rm nb}$, we obtain the maximum and minimum $\tau_{\rm nb}$s, as shown in Fig. S1(b). Although such non-binary cases occur quite often, the corresponding IETs are mostly less than 1 minute, hence having a negligible effect on the results of relevant timescales from minutes to days or even longer. The reason why there are non-binary cases might be due to the time resolution of 1 second in the empirical datasets we analyze in our work.

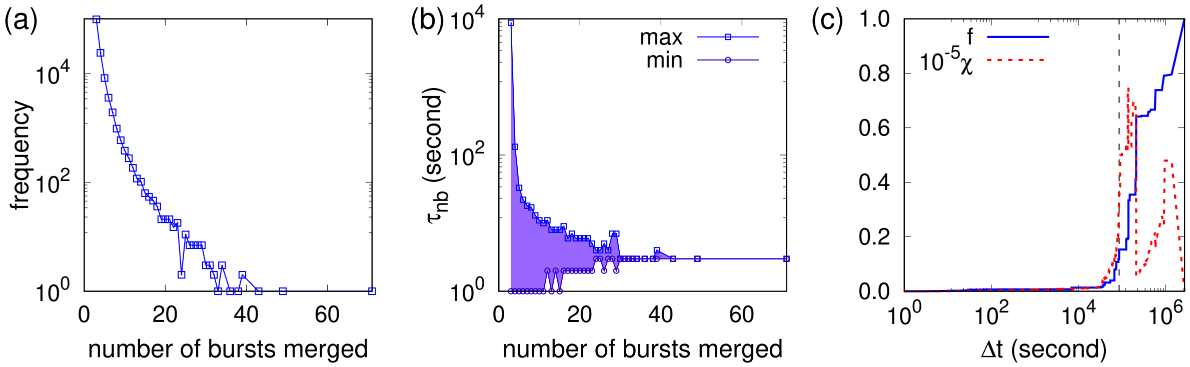

FIG. S1. Wikipedia editor 1: (a) Frequency of the number of bursts to be merged at the same time, denoted by $n_{\rm nb}$, as these bursts are separated by IETs of the same length, denoted by $\tau_{\rm nb}$, and (b) the maximum and minimum $\tau_{\rm nb}$s per each $n_{\rm nb}$. (c) The fraction of the largest burst size f in Eq. (S3) and susceptibility χ in Eq. (S4) (multiplied by 10^{-5} for clearer visualization) as functions of Δt for the percolation analysis.

To derive the binary tree structure from such cases, we repeat the binary merging of two consecutive bursts until all these bursts are merged into one burst. For example, let us denote the burst sizes of three consecutive bursts separated by the same IETs as $\{b_1, b_2, b_3\}$. These bursts can be merged in two ways: One is to first merge b_1 and b_2, and then $b_1 + b_2$

is merged with b_3. The other is to first merge b_2 and b_3, and then $b_2 + b_3$ is merged with b_1. One of these two ways is chosen at random for deriving the binary tree structure.

II. RESULTS OF OTHER WIKIPEDIA EDITORS, TWITTER USERS, AND HEALTHY SUBJECTS

In addition to the quantities discussed in the main text, we also propose two new quantities for characterizing structural properties of burst trees, namely, the memory coefficient between sibling bursts M_{lr} and the asymmetry a_u. As for M_{lr}, we consider only the pairs of sibling nodes in $C_{\Delta t}$, i.e., those sharing the same parent node. We denote the set of those siblings by $S_{\Delta t} \equiv \{(v,w)|v,w \in C_{\Delta t},\ (u,v,w) \in \mathcal{G}\}$. Then we suggest a novel definition of the memory coefficient between sibling bursts for a given Δt as follows:

$$M_{lr} \equiv \frac{1}{|S_{\Delta t}|} \sum_{(v,w)\in S_{\Delta t}} \frac{(b_v - \mu_l)(b_w - \mu_r)}{\sigma_l \sigma_r}, \tag{S1}$$

where μ_l (μ_r) and σ_l (σ_r) respectively denote the average and standard deviation of burst sizes of all the left (right) children in $S_{\Delta t}$. Positive M_{lr} implies a tendency of big (small) bursts to be merged with big (small) ones. The opposite tendency can be observed for the negative M_{lr}, while $M_{lr} = 0$ indicates the absence of correlations between sibling bursts.

Then we define for each internal node u the asymmetry between its two children with burst sizes b_v and b_w as

$$a_u \equiv \frac{b_w - b_v}{b_w + b_v}\,. \tag{S2}$$

If $b_w = b_v$, one gets $a_u = 0$. If $b_w \gg b_v$, we have $a_u \approx 1$, while $a_u \approx -1$ for $b_w \ll b_v$. For a given Δt, we take an average of a_u over the nodes whose associated IETs are the same as Δt, and denote it by $\langle a_u \rangle$.

We apply our burst-tree decomposition method to other editors in the English Wikipedia dataset. In particular, we analyze the edit sequences of the 20 most active editors (including the editor 1) in terms of their edit numbers, as summarized in Fig. S2. We find the similar, universal behaviors for all active editors such as heavy-tailed burst size distributions for a wide range of Δt, overall positive values of M_b and M_{lr} for several decades of Δt, overall increasing behavior of the diagonal cross-section of the merging kernel, $K(b,b)$, and $\langle a_u \rangle$ that are negligible for small Δt, but largely fluctuating for large Δt.

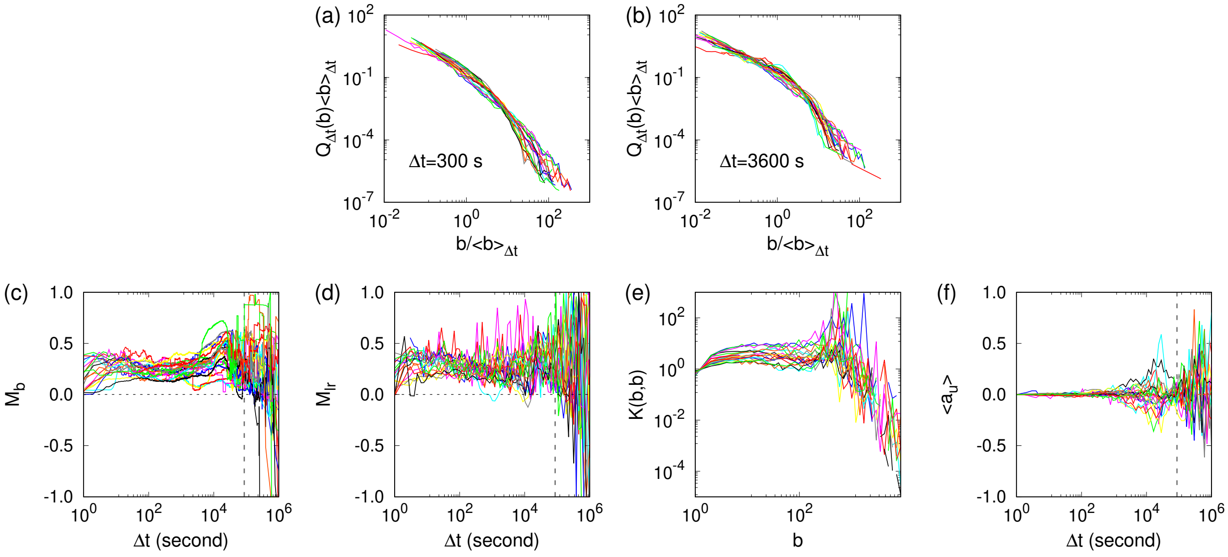

FIG. S2. Wikipedia's top-20 most active editors: (a, b) Burst size distributions for $\Delta t = 300$ and 3600 seconds, (c) memory coefficients between two consecutive burst sizes M_b, (d) memory coefficients between two sibling bursts M_{lr}, (e) diagonal cross-sections of the merging kernel $K(b, b)$, and (f) the averaged asymmetries $\langle a_u \rangle$. In panels (a, b), $\langle b \rangle_{\Delta t}$ is the average burst size for a given Δt. Vertical dashed lines in panels (c, d, f) denote $\Delta t = 1$ day.

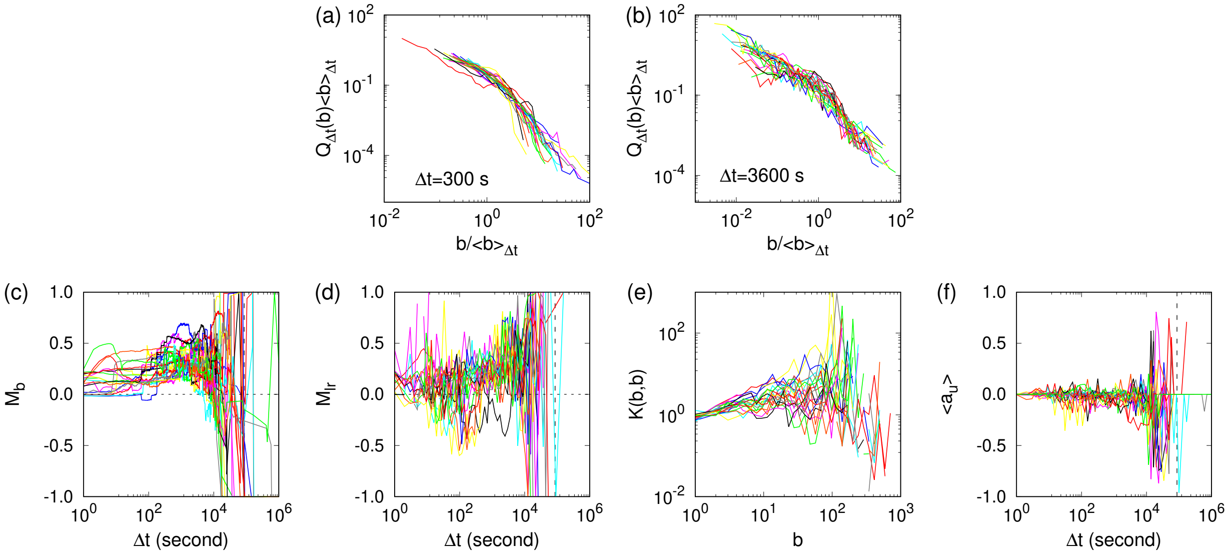

FIG. S3. Twitter's top-20 most active users. All notations are the same as in Fig. S2.

We also analyze the tweet sequences of the 20 most active Twitter users (including the user 1) from the Twitter dataset and the heartbeat time series of 20 healthy subjects (including the subject 1), as shown in Figs. S3 and S4, respectively. We find overall similar patterns

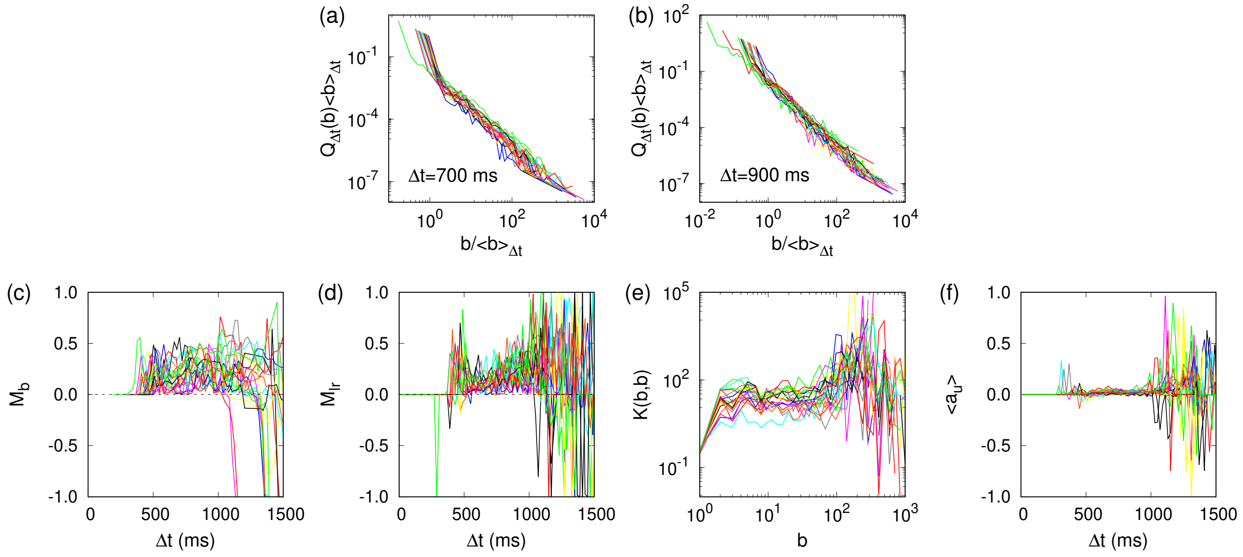

FIG. S4. Heartbeat time series of 20 healthy subjects. All notations are the same as in Fig. S2.

to those found in the Wikipedia editors, except for the M_{lr} and $\langle a_u \rangle$ in the case with Twitter users. Overall larger fluctuations are observed in Twitter dataset, probably because of relatively smaller number of events. In the case with heartbeat time series, we find fluctuating behaviors of M_b and M_{lr}.

III. PERCOLATION ANALYSIS

The power-law burst size distributions observed in the empirical analysis can be seen as a consequence of the percolation transition. It is because the burst merging process by increasing Δt can be interpreted as the percolation process by increasing the connectivity between elements in a many-body system. To test the validity of this analogy, we measure the fraction of the largest burst f and susceptibility χ at each time window Δt: For a given Δt, we first have the sequence of bursts $C_{\Delta t}$ as defined in the main text. Then using $b_{\max} \equiv \max\{b_i | i \in C_{\Delta t}\}$, f and χ are respectively defined as follows:

$$f \equiv \frac{b_{\max}}{n+1}, \tag{S3}$$

$$\chi \equiv \frac{1}{n+1} \sum_{b \in \{b_i | i \in C_{\Delta t}\} \setminus \{b_{\max}\}} b^2. \tag{S4}$$

Note that the summation in Eq. (S4) is calculated over $C_{\Delta t}$ but for the largest burst $b_{\max}$. The results for the editor 1 are shown in Fig. S1(c), where we find that the "percolating"

burst emerges only at the time window larger than ~ 1 day. In contrast, the power-law burst size distributions for the editor 1 are observed for Δt less than 1 day. These results enable us to reject the analogy between the merging process and the conventional percolation process.

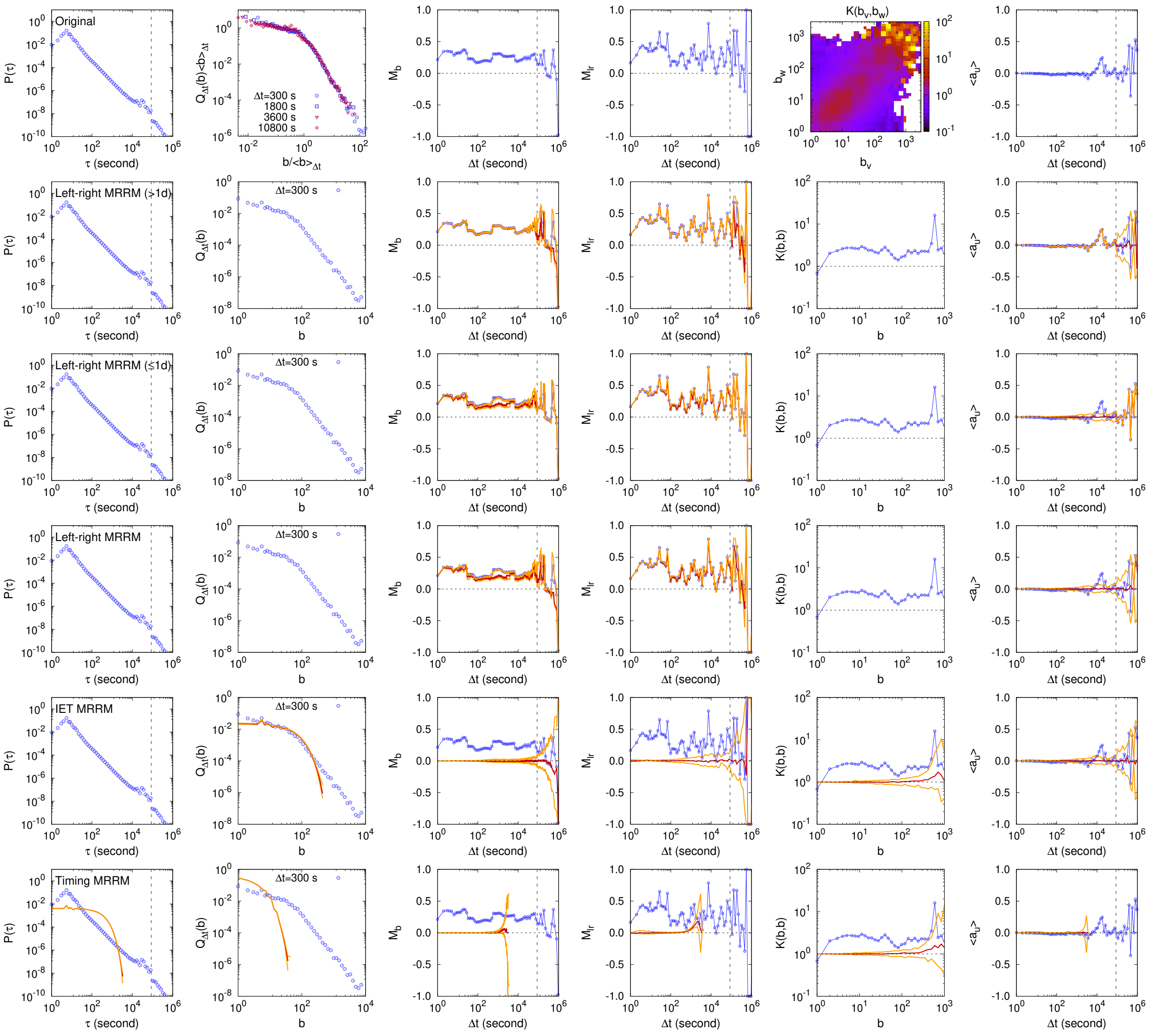

FIG. S5. Wikipedia editor 1: Complete results of several MRRMs, including the results of the original event sequence (top row). For the MRRMs used, see Table S1.

IV. COMPLETE RESULTS OF MRRMS

We present the complete results of several microcanonical randomized reference models (MRRMs) introduced in the main text for event sequences. As for the features or temporal

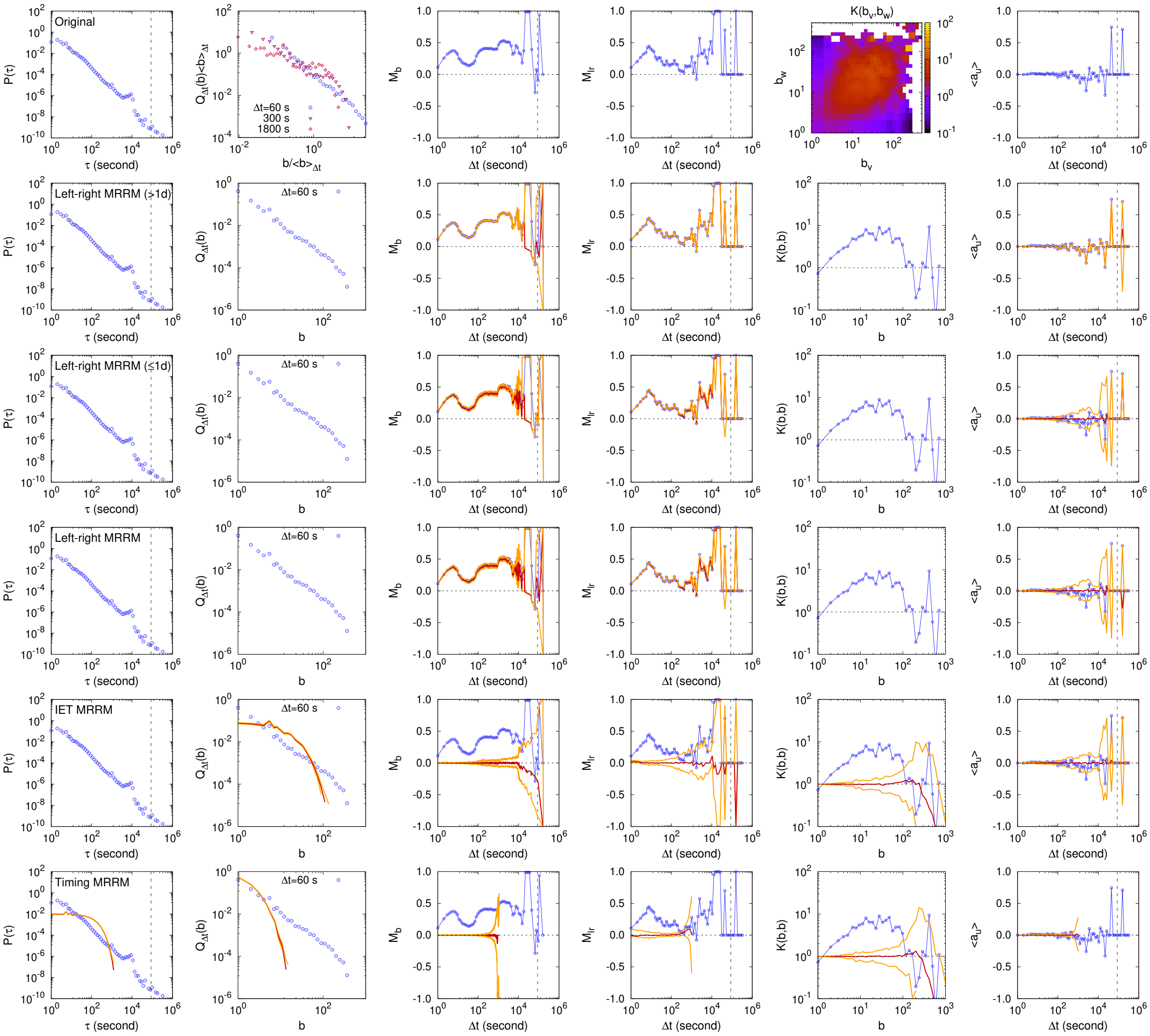

FIG. S6. Twitter user 1: Complete results of several MRRMs, including the results of the original event sequence (top row). For the MRRMs used, see Table S1.

correlations conserved in various MRRMs, we use not only the quantities discussed in the main text, i.e., $P(\tau)$, $Q_{\Delta t}(b)$, M_b, and $K(b,b)$, but also the newly introduced quantities, i.e., M_{lr} and $\langle a_u \rangle$. The list of such features is summarized in Table S1.

We apply the MRRMs to the event sequences for the editor 1 of Wikipedia (Fig. S5), for the user 1 of Twitter (Fig. S6), for the subject 1's heartbeat time series (Fig. S7), and for the JUNEC (Fig. S8). For the Wikipedia editor 1 and Twitter user 1, one can also introduce variants of the left-right MRRM by limiting the shuffling only to internal nodes with IETs larger (or smaller) than a fixed timescale of 1 day, aiming to destroy the left-right structure

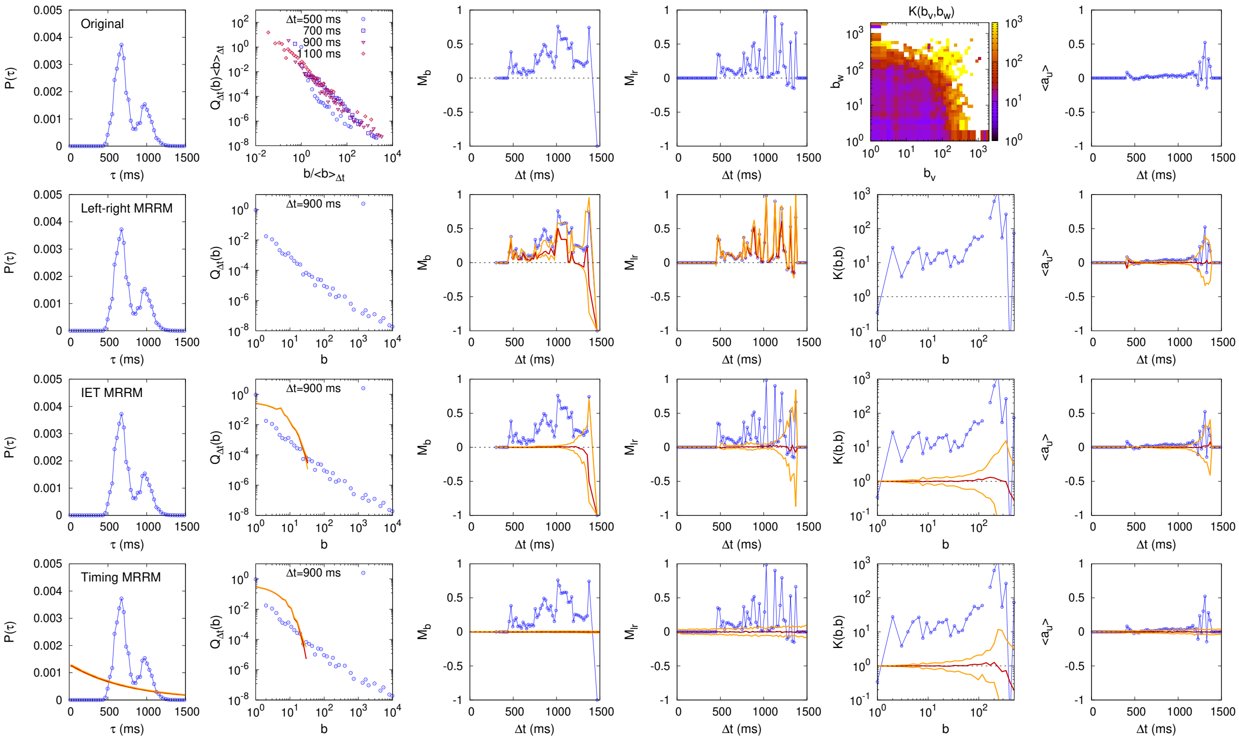

FIG. S7. Heartbeat of the healthy subject 1: Complete results of several MRRMs, including the results of the original event sequence (top row). For the MRRMs used, see Table S1.

TABLE S1. Features or temporal correlations conserved in various microcanonical randomized reference models (MRRMs). The “original” data trivially retains all features.

MRRM	$P(\tau)$	$Q_{\Delta t}(b)$	M_b	M_{lr}	$K(b,b)$	$\langle a_u \rangle$
Original	✓	✓	✓	✓	✓	✓
Left-right shuffled	✓	✓			✓	
IET shuffled	✓					
Timing shuffled						

for the timescale longer (or shorter) than 1 day. The results of these variants are included in Figs. S5 and S6.

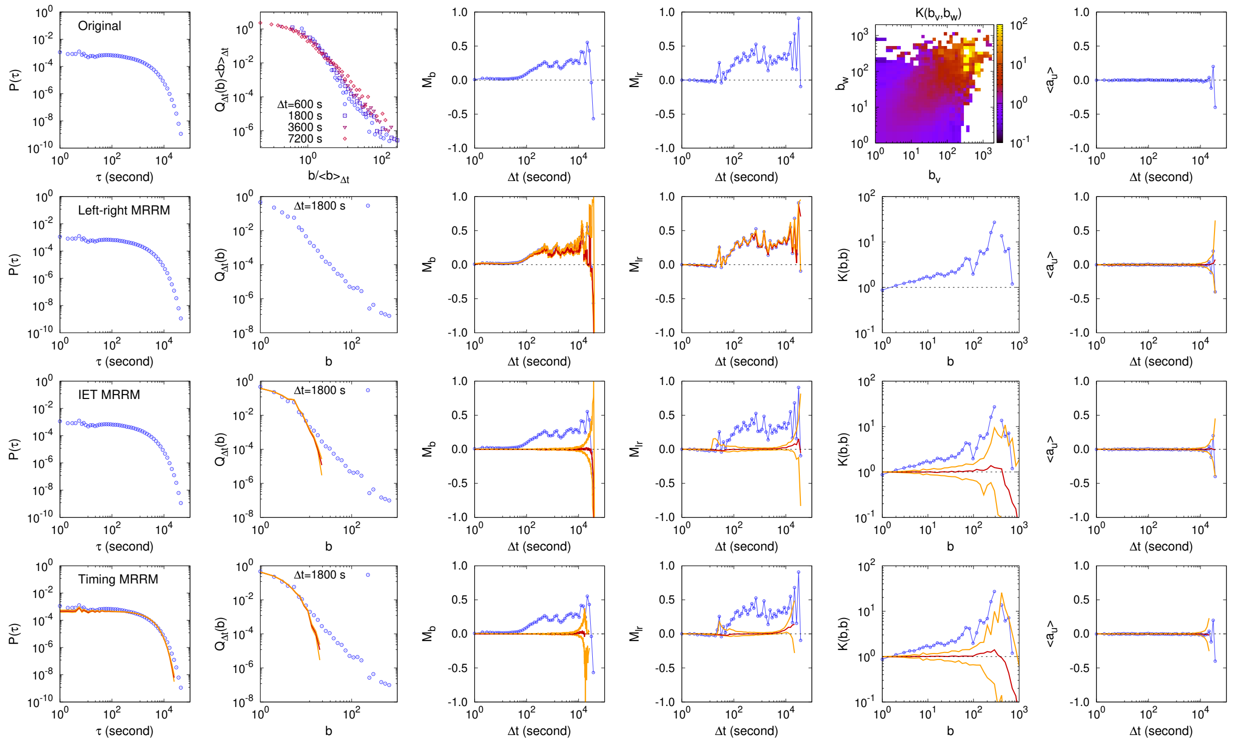

FIG. S8. JUNEC: Complete results of several MRRMs, including the results of the original event sequence (top row). For the MRRMs used, see Table S1.

V. OTHER KERNEL-BASED MODELS

In addition to the model kernel used in the main text, we test two other functional forms:

$$K(b,b') = \left[1 + c_1(\ln b + \ln b')^2\right]\left[1 + c_2 e^{-(\ln b - \ln b')^2/c_3}\right], \tag{S5}$$

$$K(b,b') = \left\{1 + c_1[(\ln b)^2 + (\ln b')^2]\right\}\left[1 + c_2 e^{-(\ln b - \ln b')^2/c_3}\right], \tag{S6}$$

where c_1, c_2 are nonnegative parameters, and c_3 is a positive parameter. For the simulations, we commonly use the same IET distribution with $\alpha = 1.8$ in Eq. (4) in the main text, to generate 100 event sequences of $n = 10^5$ for each case.

Similar to the case in the main text, we study the case with the model kernels in Eqs. (S5) and (S6) with parameter values of $(c_1, c_2, c_3) = (3, 100, 4)$, $(0, 100, 4)$, $(3, 0, 4)$, and $(3, 100, 1)$. In Figs. S9 and S10, we find the qualitatively similar results as in the case with the model kernel in the main text, enabling us to conclude that to get the heavy-tailed or power-law burst size distributions for several values of Δt and overall positive values of M_b and M_{lr} for a wide range of Δt simultaneously, both preferential and assortative mixing structures

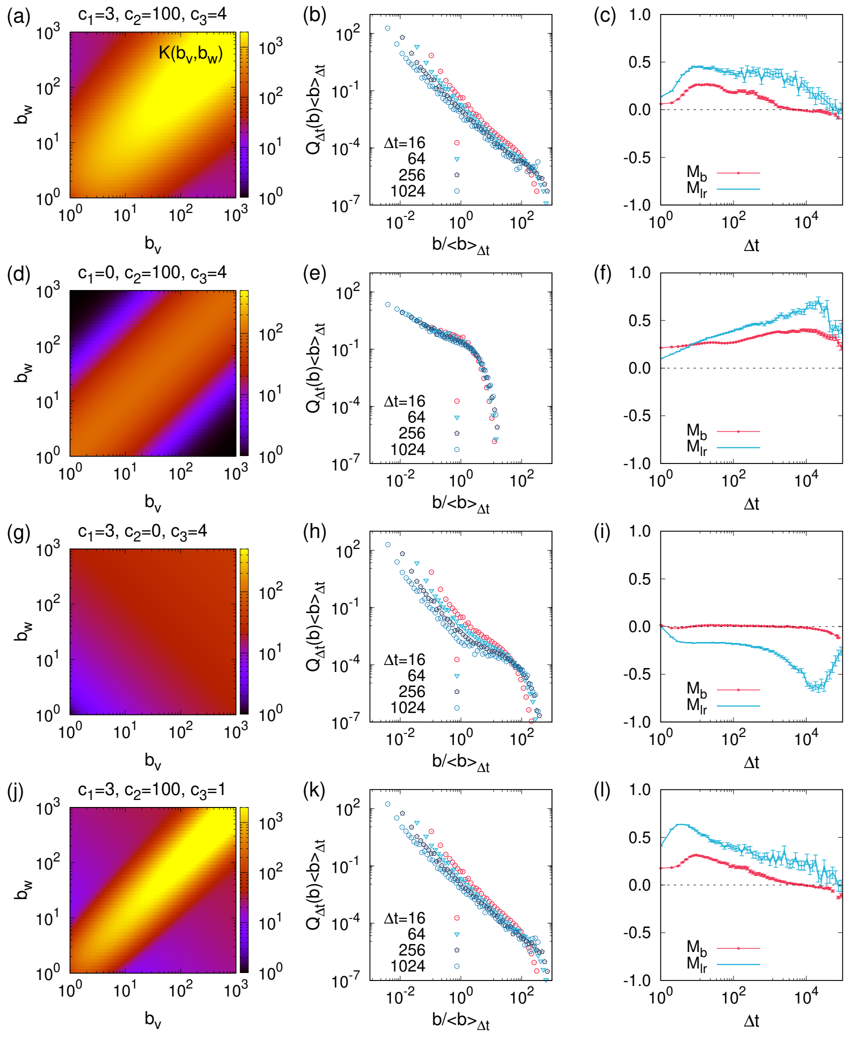

FIG. S9. Simulation results using the model kernel in Eq. (S5) with various combinations of parameter values, together with the IET distribution $P(\tau) \propto \tau^{-\alpha}$ with $\alpha = 1.8$ in Eq. (4) in the main text. The model kernels used are presented as a heatmap in left panels, while the center and right panels show the burst size distributions as well as M_b and M_{lr} obtained from 100 event sequence of $n = 10^5$. The error bars denotes the standard errors.

of bursts ($c_1 > 0$ and $c_2 > 0$) are necessary for guaranteeing the increasing diagonal $K(b, b)$ and the higher profile of the diagonal part than the off-diagonal part of $K(b_v, b_w)$.

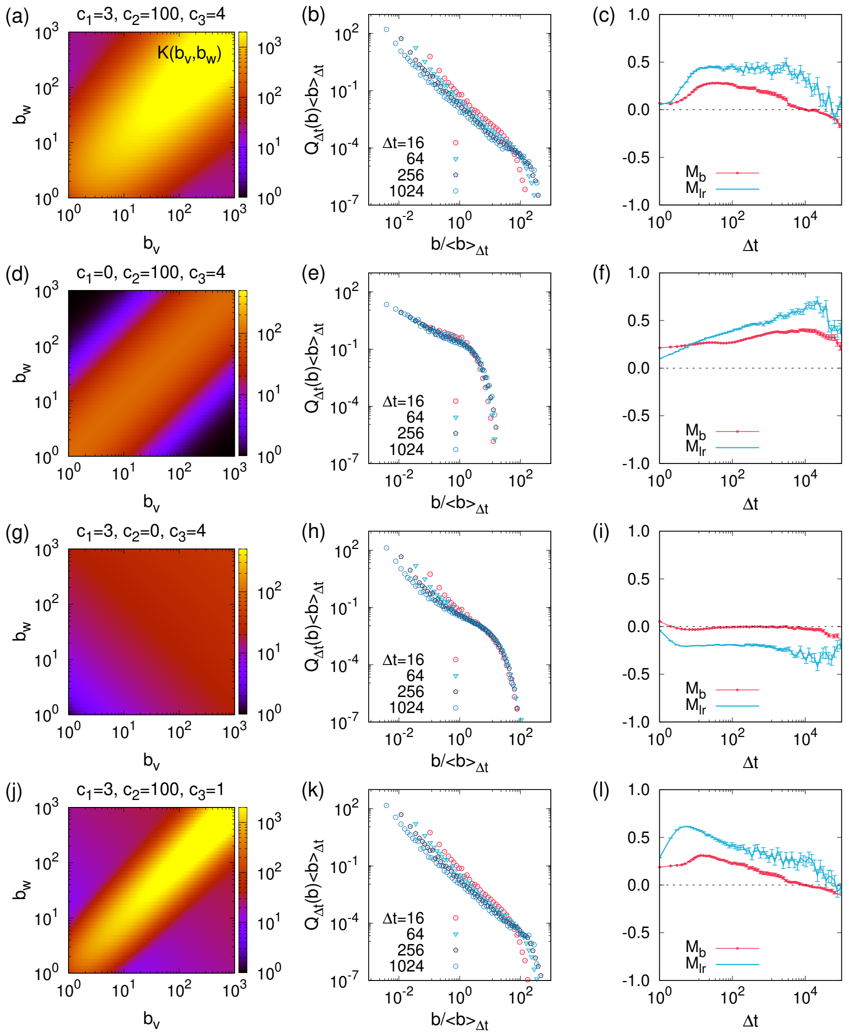

FIG. S10. Simulation results using the model kernel in Eq. (S6) with various combinations of parameter values, together with the IET distribution $P(\tau) \propto \tau^{-\alpha}$ with $\alpha = 1.8$ in Eq. (4) in the main text. The model kernels used are presented as a heatmap in left panels, while the center and right panels show the burst size distributions as well as M_b and M_{lr} obtained from 100 event sequence of $n = 10^5$. The error bars denotes the standard errors.
